# Medical Resource Management in Emergency Hierarchical Diagnosis and Treatment Systems: A Research Framework

**DOI:** 10.3390/healthcare12131358

**Published:** 2024-07-08

**Authors:** Li Luo, Renshan Zhang, Maolin Zhuo, Renbang Shan, Zhoutianqi Yu, Weimin Li, Peng Wu, Xin Sun, Qingyi Wang

**Affiliations:** 1 Business School, Sichuan University, Chengdu 610065, China; 2 School of Finance and Trade Management, Chengdu Industry & Trade College, Chengdu 611731, China; 3 School of Management, Hangzhou Dianzi University, Hangzhou 310018, China; 4 West China Hospital, Sichuan University, Chengdu 610041, China

**Keywords:** emergency medical resource management, emergency hierarchical diagnosis and treatment system, major public health emergencies

## Abstract

The occurrence of major public health crises, like the COVID-19 epidemic, present significant challenges to healthcare systems and the management of emergency medical resources worldwide. This study, by examining the practices of emergency medical resource management in select countries during the COVID-19 epidemic, and reviewing the relevant literature, finds that emergency hierarchical diagnosis and treatment systems (EHDTSs) play a crucial role in managing emergency resources effectively. To address key issues of emergency resource management in EHDTSs, we examine the features of EHDTSs and develop a research framework for emergency resource management in EHDTSs, especially focusing on the management of emergency medical personnel and medical supplies during evolving epidemics. The research framework identifies key issues of emergency medical resource management in EHDTSs, including the sharing and scheduling of emergency medical supplies, the establishment and sharing of emergency medical supply warehouses, and the integrated dispatch of emergency medical personnel. The proposed framework not only offers insights for future research but also can facilitate better emergency medical resource management in EHDTSs during major public health emergencies.

## 1. Introduction

Major public health emergencies are characterized by their sudden occurrence, severe impact on public health, and involvement of various types of crises, such as the epidemics of SARS, H1N1, and COVID-19. The COVID-19 epidemic broke out at the beginning of 2020 and influenced over 200 countries worldwide. This pandemic has not only caused significant economic losses worldwide, but has also posed a serious threat to human life safety. As of May 2024, there had been more than 775 million confirmed cases of COVID-19 worldwide and 7.04 million deaths. Due to the COVID-19 epidemic, many countries faced practical challenges such as shortages of emergency medical personnel, insufficient emergency medical supplies (e.g., medical protective masks, clothing, goggles), and highly limited emergency medical supply production capacity [1]. Consequently, rational management of limited emergency medical resources, i.e., emergency medical personnel and medical supplies, plays a crucial role in effectively preparing for and responding to major public health emergencies [2,3].

In the global battle against the epidemic, various countries have taken different measures to ensure a more effective and equitable use of limited emergency medical resources [4]. In particular, the practices of emergency medical resource management in representative countries such as China, the United States, Singapore, and Germany demonstrate that the allocation of medical resources based on a hierarchical diagnosis and treatment system is beneficial for the effective control of epidemics. Since September 2015, a hierarchical diagnosis and treatment system has been gradually implemented in China [5,6]. Healthcare coalitions, as a specific type of hierarchical diagnosis and treatment systems, achieved significant success in controlling regional outbreaks of the COVID-19 epidemic in China [7,8]. Taking advantage of healthcare coalitions, more than 4 million medical personnel across the country have collaborated, alleviating the burden on large hospitals by implementing hierarchical diagnosis and treatment, which involves primary screening, diagnosis, and referral [9]. This further emphasizes the practical importance of managing emergency medical resources within a framework of hierarchical diagnosis and treatment.

**Figure 1:**
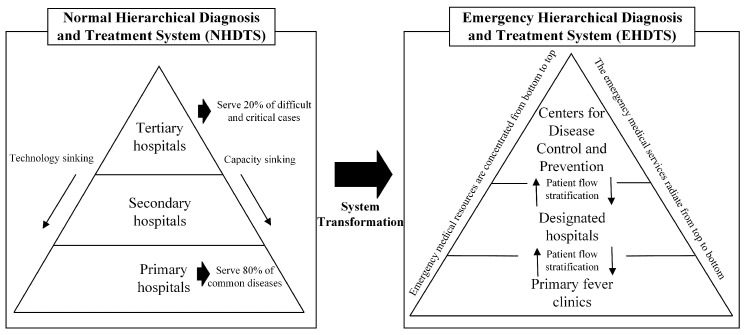
The system transformation from NHDTS to EHDTS.

Due to the uncertain, dynamic, and complex nature of public health emergencies, the management of medical resources in hierarchical diagnosis and treatment systems is challenging. Therefore, based on the medical resource management practices in China, the United States, Singapore, and Germany, and reviewing the relevant theories and literature, we develop the concept of an emergency hierarchical diagnosis and treatment system (EHDTS), which aims to provide a rapid and effective emergency response by transforming the role of a normal hierarchical diagnosis and treatment system (NHDTS). As shown in Figure 1, an NHDTS includes tertiary hospitals serving 20% of difficult and crucial cases, secondary hospitals, and primary hospitals serving 80% of common diseases. In an NHDTS, technology and capacity sink from tertiary hospitals to primary hospitals. When public health emergencies occur, the NHDTS transforms into an EHDTS with patient flow between centers of disease and prevention, designated hospitals, and primary fever clinics. In an EHDTS, emergency medical resources are concentrated from bottom to top, and emergency medical services radiate from top to bottom. Based on the EHDTS, this study develops a framework for emergency medical resource management and discusses some key future research issues. This study offers guidance and future research ideas on the establishment of an effective EHDTS based on an NHDTS, improving the health and medical services of an established EHDTS, and enhancing emergency medical resource management for major public health emergencies in the future.

The remainder of this article includes four sections: Section 2 discusses the practices of emergency medical resource management in four representative countries. Section 3 conducts a brief literature review. Section 4 presents a research framework for emergency medical resource management in an EHDTS. Section 5 draws conclusions.

## 2. Practices of Emergency Medical Resource Management in Representative Countries

This section investigates the practices of managing emergency medical resources in China, the United States, Singapore, and Germany. These practices serve as a practical foundation for developing the concept of the EHDTS and identifying important research topics in this area.

### 2.1. China

During the initial stage of the COVID-19 outbreak, the Chinese central government established the principle of hierarchical diagnosis and treatment of classified COVID-19 patients and required that all provinces pool resources, leverage healthcare coalitions to strengthen hierarchical diagnosis and treatment with limited medical resources. A total of about 15,000 fever clinics and 2092 designated hospitals were established in China to control the epidemic [2]. As one of the areas hardest hit by the epidemic, Hubei province adopted a county–township–village three-level emergency medical service model to carry out epidemic prevention and control to strengthen up–down linkage and resource sharing among designated hospitals, townships, and community clinics [3]. This anti-epidemic mode is promoted and implemented under the technical guidance of disease control agencies and includes specific measures such as deploying centralized isolation points and transforming designated healthcare service institutions to effectively carry out patient triage, referral, and regular health services. In particular, on 25 February 2020, Wuhan, the capital of Hubei province, established an EHDTS that included 61 outpatient medical facilities and 48 designated hospitals. The number of designated hospitals and available sick beds increased rapidly from 23 and 6754 on 1 February to 48 and 24,378, respectively, providing strong support for the prevention and control of the COVID-19 epidemic [7]. According to the EHDTS in Wuhan, each community is responsible for comprehensive screening of patients with fever and referring suspected COVID-19 patients to community healthcare service centers for further diagnosis and classification [5]. Individuals diagnosed with COVID-19 will be directed to specific medical facilities for appropriate care, and infected patients who exhibit mild and severe symptoms will be referred to different medical facilities. To effectively alleviate pressure on medical facilities, Wuhan first renovated 16 temporary shelter hospitals to provide 12,666 sick beds for mild-symptom COVID-19 patients [6]. Huoshen Mountain Hospital and Leishen Mountain Hospital were newly built to offer about 2500 sick beds for the care of severe and critical COVID-19 patients [5]. However, the number of infected patients in Wuhan reached a peak of 38,020 on 18 February, leading to bed waste in designated hospitals and temporary shelter hospitals after 29 February [3].

In addition, the other provinces dispatched emergency medical teams to assist Hubei province following the arrangements of the central government. A total of 346 national medical teams with approximately 42,000 medical personnel were dispatched to Hubei province, of which a total of 315 medical teams with approximately 35,611 medical personnel supported Wuhan [8]. Specifically, a total of more than 1400 and 2900 medical personnel were dispatched to Huoshen Mountain Hospital and Leishen Mountain Hospital, respectively. A total of more than 8000 medical personnel supported temporary shelter hospitals. To address the shortage of emergency medical supplies such as N95 masks and protective clothing, the central government established a policy aimed at thoroughly evaluating the availability and demand of emergency medical supplies and coordinating the transshipment of emergency medical supplies throughout the country. As a result, meticulous management of key emergency medical supplies was put in place throughout China, facilitating rational distribution of these supplies among hospitals of varying regions and levels. In particular, the other provinces delivered approximately 8 million pieces of medical protective clothing, 18,000 ventilators, and 1.65 million medical masks or eye shields to Hubei province, and most of the supported supplies were delivered to Wuhan considering key factors such as the type and number of patients served and the supply shortages in various emergency medical facilities [7]. In Wuhan, since a large number of suspected and confirmed patients with COVID-19 entered medical facilities in the early stage, normal outpatient services were stopped for a while. Some Wuhan hospitals started slowly reintroducing regular outpatient services after 5 March 2020, and before that date, only care for emergency cases such as heart attacks and trauma was offered [10].

From the case of China, we find that the emergency medical resource management of China included the following key measures: (1) the rapid development of an EHDTS based on its existing NHDTS; (2) centralized decision-making to facilitate coordinated epidemic control and transshipment of emergency medical resources in various regions; (3) enhancing emergency medical resource support in different provinces by establishing various policies and regulations in a timely manner. However, the anti-epidemic practices of China also face challenges such as imbalance between the supply and demand of medical resources of various types of patients, the waste of emergency medical resources caused by not accurately forecasting the evolution of epidemics, and the lack of decision-aiding tools based on multidisciplinary theories and techniques such as operations research, emergency management, public health, and artificial intelligence.

### 2.2. United States

In the early stage of the COVID-19 epidemic, anti-epidemic practices in the U.S. were mainly led by state governments and differed from state to state. In the three-level system (including the federal disease control and prevention system, the state or local hospital emergency preparedness systems, and the metropolitan medical emergency systems) for public health emergencies in the U.S., the states activate health emergency operation centers, deploy temporary medical stations and temporary hospitals to deal with the epidemic [11]. With the established U.S. EHDTS, suspected or mild-symptom COVID-19 patients are typically managed through home isolation and remote medical assistance, and severe cases are referred to hospitals for advanced care. During the COVID-19 epidemic, the number of infected people in the U.S. exceeded 103 million, and neither bed resources nor medical personnel could meet the sharply increased demand, suggesting that the U.S. faced a severe shortage of medical resources during the COVID-19 epidemic [12]. To deal with the problem of insufficient medical resources, the U.S. federal and local governments launched a series of measures to optimize resource allocation and reserve emergency supplies at three levels, i.e., the national level, regional/state level, and local level. In particular, the epidemic was more serious in New York state, and the sharply increased patient numbers led to challenges, such as shortages of medical supplies and personnel, as well as inadequate hospital bed capacity [13]. To tackle the challenges, New York state put forward various measures to improve its diagnostic and treatment service capacity and streamline the distribution of medical resources, such as converting the Javits Convention Center and the Queens Racetrack into temporary shelter hospitals with 2900 and 1000 beds, respectively; designating the U.S. Navy’s 1000-bed hospital ship for non-COVID-19 patients so that hospitals in New York state could allocate more resources for COVID-19 patients; broadening collaboration with emergency medical supply providers for items like masks and goggles; activating over 60,000 volunteers, including retired medical professionals, to assist major hospitals in New York City [14]. However, the U.S. did not effectively control the epidemic in the early stage. The halting and postponement of non-COVID-19 medical services in numerous locations across the U.S. increased the mortality risk for non-COVID-19 patients. For instance, the majority of healthcare facilities in New York state delayed non-urgent surgeries, with clinics functioning at reduced capacity, leading to a significant decrease in the number of non-COVID patients receiving treatment for diseases such as heart disease, stroke, and cancer.

We find that the management of emergency medical resources in the U.S. includes key measures such as (1) the deployment of a three-level system for emergency supply reservation and allocation, (2) transforming large venues and facilities to temporary emergency medical facilities to expand the capacity of emergency medical service, and (3) quickly mobilizing medical volunteers to supplement emergency medical manpower. The U.S. practice also faces some challenges. First, coordinated cross-state emergency medical resource management can be strengthened. Second, coverage of emergency medical supplies and services can be improved for some populations, such as low-income COVID-19 patients. Third, compulsory public requirements for epidemic control can be implemented to reduce the growth rate of emergency medical resource demand early after an epidemic outbreak. Lastly, the U.S. EHDTS can be improved to manage both patients and emergency medical resources.

### 2.3. Singapore

The Singapore government prioritizes managing the COVID-19 epidemic and mandates that individuals showing symptoms of infection must receive treatment at specified hospitals. After the outbreak of the COVID-19 epidemic, the Singapore government quickly established an emergency medical service system based on community clinics and general hospitals, and launched the Public Health Preparedness Clinic (PHPC) system, which includes 969 public health prevention clinics. The PHPC system serves as a platform for the initial diagnosis and triage of patients exhibiting symptoms of respiratory infections. Additionally, it enables numerous general clinics in the community to promptly and efficiently address challenges like inadequate medical resources and increased medical demand, in alignment with the unified directives of the Singapore health department. The emergency clinics within the PHPC system follow standard protocols for diagnosing, treating, reporting, transferring, and isolating suspected patients, which helps to reduce instances of overlooked diagnosis and protects the limited medical resources of major general hospitals from being overwhelmed. Patients who receive a COVID-19 diagnosis within the PHPC system will be systematically organized and moved to larger hospitals with available emergency medical resources for additional care. To alleviate the pressure on large hospitals, the Singapore government also transformed its National Center for Infectious Diseases (NCID) into the Singapore version of the Huoshen Mountain Hospital to centralize the treatment of confirmed COVID-19 patients and increased the number of NCID beds from 330 to 500 [15]. Moreover, the Singapore government has introduced the SG Healthcare Corps plan to support medical personnel combating COVID-19 and has readied more than 3000 medical personnel for anti-epidemic work. However, due to the relatively low coverage of Singapore’s medical service system for foreign workers, Singapore has experienced epidemic rebound events such as cluster infections and community transmission among foreign workers. To strengthen the epidemic control, the Singapore government emulated China’s approach by building temporary shelter hospitals, transforming its Expo Center, Changi Convention and Exhibition Center, and Tanjong Pagar Container Terminal into temporary community isolation facilities which could accommodate more than 40,000 patients with mild symptoms [16]. Singapore also implemented remote consultation technology to offer nursing services 24/7 at community isolation facilities, thereby alleviating the pressure on front-line medical resources needed to combat the epidemic.

From the case of Singapore, we find that (1) the anti-epidemic practice of Singapore is mainly based on an EHDTS composed of community clinics, large general hospitals, the NCID, and temporary isolation points; (2) the PHPC system plays a key role, and Singapore effectively controlled the epidemic in its early stage by leveraging the PHPC system’s capacity to swiftly transition between different service statuses and to offer emergency (routine) medical services to COVID-19 (general) patients. The anti-epidemic practice of Singapore unveils the following challenges. First, to ensure that some minor populations, such as foreign workers, are adequately covered, emergency medical service capacity should be expanded with limited emergency medical resources. Second, developing emergency plans and taking advantage of existing NHDTSs is challenging but fundamental to better prepare and utilize limited emergency medical resources. Third, it is vital to improve emergency medical service capacity and population coverage by dynamically allocating limited emergency medical resources that match the evolution of epidemics.

### 2.4. Germany

Before the first confirmed case, the German government, foreseeing the global spread potential of COVID-19, collected and analyzed virus information, initiated diagnostic testing research, and developed emergency plans. Protocols for handling suspected and confirmed cases were formulated and distributed to local governments at all administrative levels. For instance, Bavaria detected Germany’s first COVID-19 case on 27 January, but as early as 21 January, its healthcare departments of various levels received directives and protocols and organized emergency medical supplies and isolation facilities accordingly [17]. Local governments quickly followed these guidelines, rapidly testing all relevant individuals after detecting and isolating confirmed cases, and providing centralized quarantine facilities for contacts. Germany adhered to the Infection Protection Act and built a national EHDTS by defining organizational structures, authorities, and responsibilities for federal, state, and local health departments. In particular, local healthcare facilities have the autonomy to design and enforce specific measures tailored to local economic and medical realities [18]. For example, following the first confirmed case in Heinsberg County, North Rhine-Westphalia, local schools and kindergartens were temporarily closed, and county officials suspended public transport despite no federal recommendation for such measures at that time [19]. Despite Germany’s relatively abundant emergency medical resources, the federal government maintained a principle of treating severe cases in hospitals while advocating home recovery for mild cases among diagnosed COVID-19 patients. Approximately 17% of the confirmed cases in Germany required hospitalization, leaving hospital bed occupancy rates around 40% [19]. Consequently, amid the rapid spread of the epidemic across Europe, Germany was able to assist countries such as France and Italy by accepting severe-symptom patients from abroad.

In general, the anti-epidemic practices of Germany suggest that (1) early and well-prepared governmental actions, rapid responses, and effective communication with local authorities help build a more effective EHDTS; (2) institutionalization of infectious disease prevention and control mechanisms, including autonomous epidemic alert systems and hierarchical response networks, can greatly facilitate emergency medical resource management; and (3) integrated management of various types of emergency medical resources is fundamental in practice. Germany also faced some challenges during the epidemic. First, decentralized decision making among local governments within the federal system made it difficult to manage limited emergency medical resources integrally. Second, behavior issues such as those related to civil liberties and privacy concerns greatly hindered some anti-epidemic measures such as social distancing and shutdowns, which are closely coupled with the management of emergency medical resources.

### 2.5. Summary

From the cases of the four countries, we find that the following problems were common in the emergency medical resource management practices of various countries during the COVID-19 epidemic. First, there was a serious shortage of emergency medical resources in medical institutions at all levels, and the allocation of medical supplies and medical personnel calls for more theoretical guidance and decision support. Second, the construction of emergency medical systems (primary medical institutions and hospitals at all levels, etc.) could have been further optimized, and the emergency transformation flexibility of normal medical service systems was relatively low. Third, dynamic emergency medical resource management mechanisms and decision-aiding tools developed by applying various theories and techniques were rarely applied in practice. Fourth, there are many research gaps between theoretical studies and practices of emergency medical resource management for epidemic control.

Moreover, we gain the following insight. First, each country has its own medical service system, which has advantages and disadvantages. EHDTSs played an important role in the prevention and control of the COVID-19 epidemic, and it was a challenge to quickly develop an effective EHDTS and manage relevant key medical resources based on existing NHDTS. Second, allocating limited medical resources considering multiple types of patients (infected and non-infected patients) has important practical significance for managing medical manpower and supplies in EHDTSs. Third, according to the evolution of the epidemic, it was urgent to dynamically manage emergency medical resources in EHDTSs. Fourth, coordinating players in an EHDTS in a timely and effective way is vital to enhance the effectiveness and fairness of medical resource management in the EHDTS.

## 3. Literature Review

This section reviews the relevant literature from three perspectives, medical resource allocation, emergency medical resource allocation, and medical resource allocation during hierarchical diagnosis and treatment. Using the keywords *Medical Resource Allocation, Hierarchical Diagnosis and Treatment System, Emergency Medical Resource Management*, and *Public Health Emergency*, we collect the relevant literature from Web of Science, one of the most comprehensive databases for global academic information. Since this literature review does not aim to be comprehensive, some relevant articles, which are not covered by the Web of Science but other databases such as PubMed/Medline, Scopus, etc., are not reviewed. The literature review helps establish the foundation for identifying key research topics and developing the research framework for medical resource management in an EHDTS.

### 3.1. Medical Resource Allocation

The allocation of limited medical resources is a key topic in healthcare management studies. Some comprehensive review articles [20,21] suggest that most studies focus on resource allocation within a single healthcare service organization. Factors such as the collaboration of multiple medical institutions [22], economic conditions [23], and fairness [24,25] are examined in the existing literature on the management of emergency medical resources. In particular, Jeon et al. [26] study socio-economic inequalities in the distribution of healthcare resources and find that few regional socio-economic related inequalities are observed in Korea. Monteiro et al. [27] investigate daily operating room time schedules under the constraints of various medical resources. Prot et al. [28] address a nurse scheduling problem with an equity objective by proposing a two-phase method consisting of first designing shifts and then assigning tasks to employees. Wang et al. [29] tackle an uncertainty-related surgery scheduling problem by developing a model that combines robust linear programming and sample average approximation. Based on real data, Zhang [6] investigate the personnel composition of different health institutions and finds that the proportion of staff in professional public health institutions is smaller than that in clinics. Through a survey, Yemeke et al. [30] investigate patients’ preferences for medical resource allocation and find that the health sector is the highest ranked sector, with 82% of the respondents selecting health as the most important sector for the government to fund. Yi et al. [31] examine ways to evaluate the effectiveness of existing medical resource management considering regional heterogeneity.

Our review of medical resource allocation studies indicates that the existing literature mainly focuses on emergency medical resource management in one hospital, and optimization studies on emergency medical resource management in an NHDTS or EHDTS, which involve multiple medical institutions, are relatively rare.

### 3.2. Emergency Medical Resource Allocation

Although the literature review [32] on emergency logistics is fruitful, there are few review studies [33] that focus on emergency medical resource allocation. In recent years, optimization studies for general emergency supply planning [34,35,36,37,38,39,40] have attracted much research effort. In comparison, studies that focus on emergency medical resource planning are still relatively rare, and they aim to develop more effective decision support tools for emergency medical resource management considering key factors of various public health events and employing methods such as mixed-integer programming [41,42], multi-objective programming [43], stochastic programming [44,45], Bayesian sequential decision making [14], and descriptive and quantitative research [13,46]. Specifically, Rachaniotis et al. [47] propose an optimization model to serve a large population with limited epidemic prevention resources. Lee et al. [48] establish an optimization model for the distribution of vaccines in the early stage of the H1N1 epidemic and suggest that children receive the highest priority. Wang et al. [49] use game theory to explore the allocation of limited medical resources between two countries after an epidemic outbreak. To address resource allocation problems between different countries, Koyuncu and Erol [50] propose a multi-objective optimization model to assess the impact of factors such as attack rates, hospitalization ratios, and death ratios. Wang et al. [51] develop a data-driven multi-objective optimization model for emergency medical resource management, and Savachkin and Uribe [52] develop a simulation optimization model for dynamic emergency medical resource allocation during pandemics.

Some studies focus on the key factors that affect the allocation of emergency medical resources, and factors such as patient classification priority [53,54], availability of human resources [55], spatial factors [56], and ethical rules [57,58,59] are investigated. Specifically, Zaric and Brandeau [60] propose a model to allocate important medical resources for epidemic control, and the impacts of budget constraints and different intervention measures are explored. Sun et al. [61] establish a model to examine the impacts of the initial number of infections on the allocation of medical resources among countries considering the existing inventories of each country. Marckmann et al. [62] find that the allocation of emergency medical resources during the COVID-19 epidemic was affected not only by patient needs and treatment preferences, but also by the clinical application standards of medical resources. He et al. [10] propose a multi-period location-allocation model which explicitly considers the spatial-temporal distribution of the testing population and the time-varying availability of various testing resources for epidemic control. To explore the impacts of random medical supply allocation strategies, Worby and Chang [63] build a medical resource allocation model considering the variability of supply and demand over time and find that the use of medical masks is an effective intervention strategy and that optimization of supply distribution is important when resources are limited. Ford et al. [64] propose an improved protocol to treat patients with acute ischemic stroke with limited emergency medical resources. Chowdhury et al. [1] show that the protective material allocation strategy is affected by the development of epidemics and short-term supply and demand.

Our literature review on emergency medical resource allocation indicates that (1) most existing studies focus on single-stage or two-stage emergency medical resource allocation, and studies on multi-stage emergency medical resource allocation are relatively rare; (2) dynamic emergency medical resource allocation considering the time variance of resource demand and supply calls for more research efforts; (3) the existing studies seldom consider the collaborative allocation of normal and emergency medical resources for better epidemic control.

### 3.3. Medical Resource Management in Hierarchical Diagnosis and Treatment Systems

To develop various decision support tools for the management of medical resources in hierarchical diagnosis and treatment systems, diverse theories and techniques such as descriptive statistics [4], mixed-integer programming [65], mixed-integer nonlinear programming [66], and game theory [67] are applied. In particular, Barr et al. [66] establish a model to develop an effective hierarchical diagnosis and treatment service network that facilitates rational allocation of patients and family physicians. Wang et al. [68] propose a two-stage stochastic emergency supply planning model to integrate pre-disaster emergency supply pre-positioning and post-disaster emergency supply transshipment and procurement in a regional healthcare coalition. Luo et al. [9] develop a two-stage stochastic programming model which deploys various types of emergency healthcare facilities before an epidemic and serves infected and non-infected patients dynamically during the epidemic. By establishing a discrete-time Markov chain, Pan et al. [69] address the problem of unreasonable allocation of medical resources at higher- and lower-level medical institutions by developing a patient appointment optimization model. Qiu et al. [70] evaluate patient flow distribution and propose a resource allocation method to improve the performance of an NHDTS. Luo et al. [71] propose a multi-period location-allocation model to deploy emergency hospitals, allocate emergency medical supplies, and manage infected patients dynamically and integrally. Wang et al. [67] apply game theory and queueing theory to study resource allocation in a two-level hierarchical diagnosis and treatment system. Wichmann and Wichmann [72] employ the Cobb–Douglas production function to study the efficiency of public health services in urban Brazil and find that human resources are vital to improving medical services.

The literature review indicates that studies on the management of medical resources in hierarchical diagnosis and treatment systems are relatively rare, and existing studies focus mainly on the impacts of the hierarchical diagnosis and treatment service system on resource allocation decisions and the key factors that influence the hierarchical diagnosis and treatment service.

In summary, our brief literature review suggests that existing studies rarely consider the establishment of an EHDTS and the associated integral allocation of medical resources in hierarchical diagnosis and treatment systems. We also find that less attention has been paid to the integrated management of medical resources (supplies and personnel) for multiple types of patients before and after specific emergencies such as large-scale epidemics.

## 4. A Research Framework of Medical Resource Management in Emergency Hierarchical Diagnosis and Treatment Systems

Based on the anti-epidemic practices of four countries and the relevant literature, we find that it is vital to transform an NHDTS into an effective EHDTS after the onset of an epidemic. We analyze the differences between NHDTS and EHDTS in terms of the characteristics of medical resource management in Table 1.

**Figure 2:**
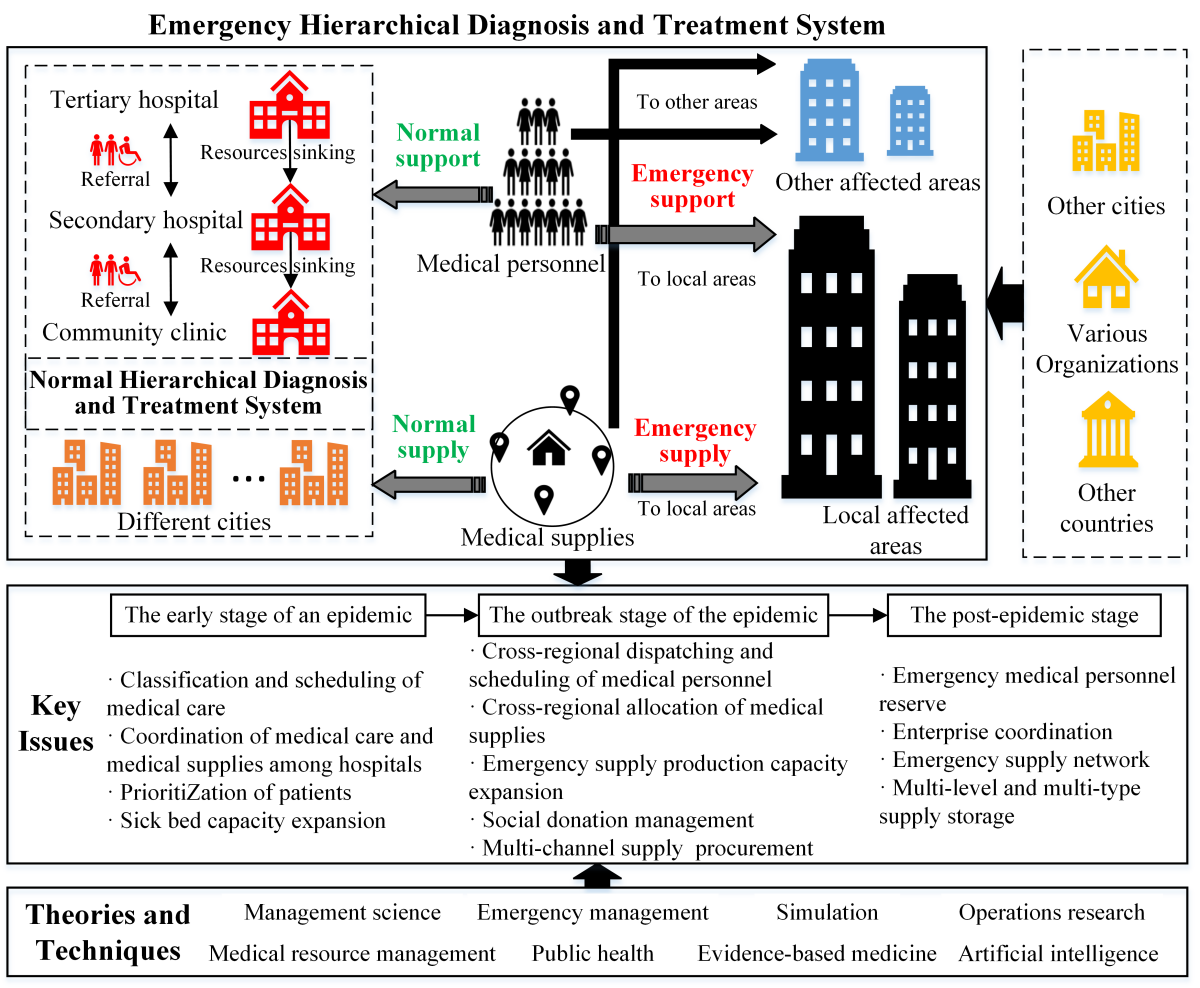
Research framework for medical resource management in an EHDTS.

In contrast to the management of medical resources in the NHDTS, the medical resource management in the EHDTS encounters more intricate circumstances, such as uncertain demand, various types of patients, high time urgency, and high supply shortage risk [32,33]. Therefore, how to establish an effective EHDTS and utilize limited key medical resources is worthy of in-depth investigation. In addition, it is crucial to analyze relationships between various types of medical resources and address resource allocation problems in EHDTS at different stages of an epidemic [40,51]. According to the literature review, we develop the research framework shown in Figure 2 to summarize key research issues of emergency medical resource management in an EHDTS.

The upper half of Figure 2 illustrates the links between the medical resource management of NHDTS and EHDTS. The dashed-line box on the left shows the NHDTS, which allows normal medical supplies (personnel) to be allocated (assigned) to different cities and enables the two-way patient referral between various types of hospital (tertiary hospitals, secondary hospitals, and community hospitals). The solid-line box represents the EHDTS, and the thick arrows represent the support (allocation) of emergency medical personnel (supplies). Figure 2 shows that (1) the construction of the EHDTS involves the existing NHDTS, which provides normal manpower support and normal medical supply, and (2) the support and allocation of emergency medical resources depend on existing resources in the EHDTS and emergency medical resources in other organizations, cities, and even countries. The lower half of Figure 2 list the key research issues related to the management of medical resources in the three stages of epidemics and the relevant theories and techniques. Specifically, in the early stage of the epidemic, key research issues such as classification and scheduling of medical care, coordination of medical care and medical supplies among hospitals, prioritization of patients, and sick bed capacity expansion can be investigated. In the outbreak stage of the epidemic, problems such as cross-regional dispatching and scheduling of medical personnel, cross-regional allocation of medical supplies, emergency supply production capacity expansion, social donation management, and multi-channel supply procurement, can be examined. In the post-epidemic stage, issues such as emergency medical human resource preparation, emergency supply coordination between medical facilities and companies, emergency supply network upgrading, and multilevel and multi-type supply storage can be studied. Moreover, studies on such research issues can benefit from an integrated application of various theories and techniques such as management science, emergency management, simulation, operations research, medical resource management, public health, evidence-based medicine, and artificial intelligence [45,65].

### 4.1. Planning of Medical Resources in Emergency Hierarchical Diagnosis and Treatment Systems in the Early Stage of an Epidemic

In the early stage of an epidemic, the epidemic impact is localized, and the number of infections is small. To quickly control the epidemic, it is necessary to effectively predict the future evolution of the epidemic, develop regional emergency medical resource allocation plans, and implement other epidemic control measures [33]. To reduce infections, patients must be triaged and isolated when necessary. To satisfy the demand of various types of patients, limited medical resources should be categorized and used with different priorities [4].

Specifically, to facilitate the assignment of medical personnel, decision makers can use statistical models, simulations, and infectious disease dynamics models (such as SIR, SEIR, and SEIHR) to predict the number and spatial distribution of patients of various types in the EHDTS, and evaluate the total demand for medical personnel based on the standard ratio of medical personnel to patient [14,21,73]. Then, mathematical programming methods can be used to optimize the scheduling of medical personnel [27,28,29,74]. Considering the medical requirements to serve various types of patients in an EHDTS, it is vital to evaluate the service capacity of medical personnel according to reality [42]. For example, limited by the total number of existing medical personnel in a healthcare coalition, medical personnel who usually serve general patients may need to take care of patients with COVID-19. Therefore, the allocation and scheduling of medical personnel that consider the demand of general patients and COVID-19 patients and incorporate multi-hospital cooperative mechanisms in the EHDTS can be key research issues [6]. Considering the time-varied patient demand and medical personnel resources, the government should adjust the ratio of medical personnel to patient flexibly and dynamically according to the evolution of the epidemic. It is also vital that the government actively coordinates the dispatch of medical personnel across different healthcare coalitions and regions [38].

To facilitate the allocation of limited medical supplies, it is worth investigating how to use limited medical supplies reasonably in the healthcare coalition to meet the needs of front-line medical personnel and various types of patients. Considering the various priorities of different types of medical personnel and patients in the EHDTS, novel medical supply location-allocation models can be built to decrease (increase) the infection rate (treatment efficiency) [45]. In addition, the emergency medical supply inventory of a hospital can be optimized based on the prediction of potential epidemic impacts by employing inventory theory [39], and coordinated medical supply transshipment between hospitals and other emergency medical service facilities in the EHDTS can be optimized to improve medical supply utilization efficiency and alleviate supply shortages [10]. By predicting the evolution of the epidemic, the government can use mathematical programming to deploy designated hospitals, prepare emergency medical service capacity, and develop emergency supply plans based on established relationship and referral standards between primary hospitals, designated hospitals, and tertiary hospitals in the EHDTS so that more infected or non-infected patients are treated timely with limited emergency medical supplies [40]. It is worth studying how the government can flexibly adjust the allocation of medical resources in the EHDTS and dynamically coordinate the transshipment of emergency medical supplies between various hospitals [51]. To avoid conflicts among member hospitals of the EHDTS, the government can apply game theory methods to achieve equilibrium decision making so that total social benefits are maximized [49]. To improve the efficiency and effectiveness of the emergency service with limited emergency medical personnel and supplies, coordinated and integrated management of medical personnel and supplies in the EHDTS can be studies as well.

### 4.2. Planning of Medical Resources in Emergency Hierarchical Diagnosis and Treatment Systems in the Outbreak Stage of the Epidemic

As the epidemic scales up and occurs in multiple regions with a fast transmission speed, the number of infection cases increases dramatically and leads to a huge demand for emergency medical resources [1,21]. At this stage, the management of emergency medical resources in an EHDTS faces more challenges due to a severe imbalance of supply–demand in time and space, which calls for a thorough investigation.

**Figure 3:**
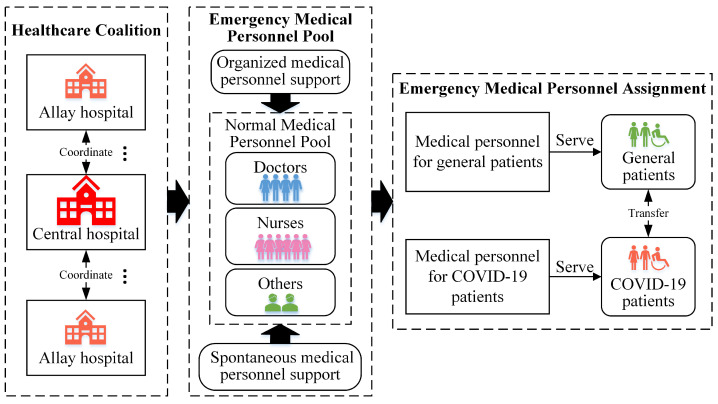
Emergency medical personnel management in healthcare coalition.

To effectively serve a large number of general patients and infected patients (COVID-19 patients) with limited medical personnel, it is vital to examine the scarcity of medical human resources in the EHDTS like a healthcare coalition, establish a pool of emergency medical personnel and dispatch various types of medical personnel integrally, as illustrated in Figure 3.

Methods like integer programming and goal programming can be employed to optimize the integrated allocation of medical personnel in an EHDTS [35]. Considering epidemic evolution, the dynamic management of medical personnel, i.e., dynamically pooling and assigning emergency medical personnel to serve various types of patients, in the EHDTS plays a key role in improving service efficiency [75]. The optimal mix ratio of various types of emergency medical personnel in the EHDTS is also worth investigating and it forms the basis for dispatching emergency medical personnel effectively [62]. Moreover, it is vital to examine how to coordinate the cross-region support of emergency medical personnel from various EHDTSs to achieve effective epidemic control on a larger scale [3].

**Figure 4:**
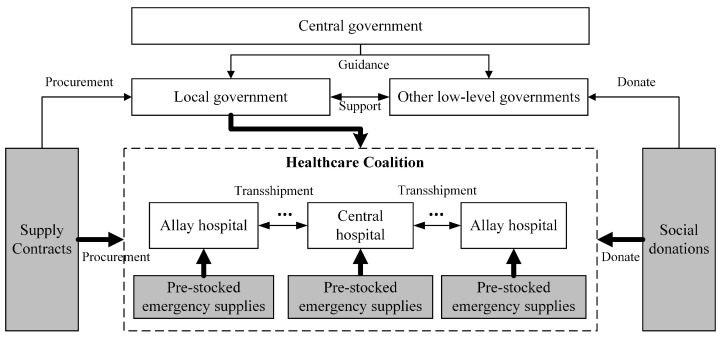
The coordinated management of emergency medical supplies.

The shortage of emergency medical supplies can be more severe during the outbreak stage of the epidemic. To reduce the shortage of emergency medical supplies, it is vital for each hospital in the EHDTS to carefully examine the availability of medical supplies and seek material support from suppliers, governments, social donations, and other medical facilities as shown in Figure 4.

Specifically, the governments of different levels should work together to coordinate emergency medical supply support between various EHDTSs, control emergency medical supply procurement, and facilitate social donations [68]. Due to high uncertainties of demand and supply at this stage, stochastic programming models and Markov decision process models can be developed to aid the multi-channel sourcing and cross-EHDTS (healthcare coalition) transshipment of emergency medical supplies [35]. It is necessary for the government to give guidance on prioritizing medical supply demand, optimize the supply network of the EHDTSs, and enhance the logistic control of rather limited emergency medical supplies by applying theories like inventory control, network flow, and emergency logistics [37]. To minimize emergency medical supply waste and logistic costs and maximize the efficiency and effectiveness of emergency medical supply utilization, the government can develop centralized supply sourcing plans that guide emergency medical supply support from various levels of governments, contracted suppliers, and social donations. Such multi-channel supply sourcing not only forms a material foundation to support the emergency response of the EHDTS but is also vital to the emergency medical supply pre-stocking and transshipment in the EHDTS [36,39,71]. To address the challenges of coordinated emergency medical supply management in a EHDTS or cross multiple EHDTSs during the epidemics, more research efforts are required from research fields like operations research, operations management, disaster management, system engineering, and emergency logistics [22].

### 4.3. Planning of Medical Resources in Emergency Hierarchical Diagnosis and Treatment Systems in the Post-Epidemic Stage

In the post-epidemic stage, the epidemic gradually become fully controlled, the number of infected patients and the medical resource shortage decrease [20,33]. To prepare for future public health emergencies, it is vital to upgrade the existing emergency medical resource management system of EHDTSs.

To increase emergency medical personnel service capacity, hospitals in an EHDTS can work together to train emergency medical personnel according to emergency response requirements and optimize emergency medical personnel structure considering cross-hospital personnel support during emergencies [75]. The member hospitals of the EHDTS should jointly build a pool of emergency medical personnel and update the responsibilities of various emergency response positions to avoid the waste of limited emergency medical personnel and to meet future emergency human resource demand effectively [76]. In addition, the government should develop policies that motivate the cooperation of schools, hospitals, and other social organizations to educate more emergency medical professionals and volunteers [77].

**Figure 5:**
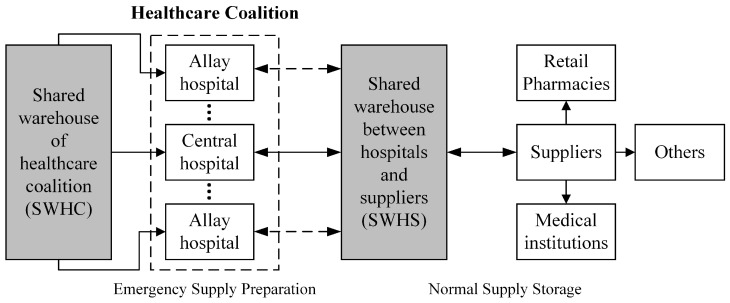
A multilevel emergency medical supply preparation system.

To strengthen emergency medical supply management in the future, it is vital to develop a multilevel emergency medical supply preparation system shown in Figure 5, which integrates normal supply storage and emergency supply preparation.

Specifically, the member hospitals of a healthcare coalition can work together to build a shared warehouse for the healthcare coalition (SWHC), which helps reduce redundant emergency medical supply storage at all member hospitals. Meanwhile, a shared warehouse between hospitals and suppliers (SWHS) can be established to reduce the inventory cost in the EHDTS under normal conditions. The SWHS can help meet the demands of various hospitals in the EHDTS quickly and reduce the risk of medical supply shortage during emergencies [1,4,21]. The multilevel emergency medical supply preparation system is a complicated system. The location, inventory level, and capacity of the SWHC and SWHS can be optimized by predicting emergency medical supply demands and applying theories and techniques such as inventory control, network flow, emergency logistics, and mathematical programming [14,20,37]. In addition, the government can establish an emergency medical supply preparation system that includes centralized official storage and decentralized storage such as community reserves, family reserves, and production reserves of enterprises, and game theory can be applied to achieve a balance of interests among the government, companies, and families [49].

## 5. Conclusions

Based on the anti-epidemic practices in China, the United States, Singapore, and Germany during the COVID-19 epidemic and the brief literature review, this study analyzes the role of EHDTS in the prevention and control of epidemics and finds that the development of an EHDTS based on an existing NHDTS is vital for the effective management of emergency medical resources for epidemic control. However, there are still many problems and challenges in the management of emergency medical resources in an EHDTS. We analyze the characteristics of an EHDTS and further propose a research framework specifically aimed at facilitating the management of emergency medical resources in an EHDTS. The research framework places particular emphasis on the effective management of emergency medical personnel and supplies during epidemics. The framework highlights key research issues such as the coordination and allocation of emergency medical supplies, the establishment and sharing of emergency medical supply warehouses, and the coordinated assignment of emergency medical personnel. The developed framework not only provides valuable guidance for future research, but also outlines key issues for better management of emergency medical resources in an EHDTS.

This study has three main limitations, which can be addressed in the future. First, this study only analyzes the anti-epidemic practices of four representative countries, three of which are developed countries. It would be interesting to analyze the anti-epidemic practices of more countries, especially developing countries, and to build a more general EHDTS framework and draw conclusions that can be applied in various countries. In addition, our case analyses is limited by focusing on some specific regions in China (Wuhan city) and the U.S. (New York state). Thus, it is important to enrich the case analyses based on more realistic data and examples from representative countries. Second, our literature review can be greatly improved in the future. More relevant studies from other databases, such as PubMed/Medline and Scopus, can be included to show the big picture of the latest studies on emergency medical resource management in an NHDTS or EHDTS. An in-depth review of the literature can also be conducted to emphasize key management issues and research challenges related to the management of various types of emergency medical resources, such as medical materials, emergency medical human resources, and emergency medical facilities, within an NHDTS or EHDTS. Third, more real-world data should be collected and analyzed to support future studies on emergency medical resource management in an EHDTS. Data on regional emergency medical resource supply and demand before and after an epidemic are crucial to revealing supply and demand patterns, which are the basis for improving emergency medical resource management in an NHDTS or EHDTS and the key to conducting real-world case studies in the future. 

## Figures and Tables

**Table 1:** Comparison of medical resource management in NHDTS and EHDTS.

Features	NHDTS	EHDTS
Demand predictability	Easy, high certainty	Hard, high uncertainty
Relevant people	General patients	General patients, infected patients
	Local medical personnel	Local and non-local medical personnel
Supply shortage risk	Low	High
Time urgency	Low	High
Inventory	Normal, relatively short term	Emergency, relatively long term
Distribution	Regular, no delay	Flexible, delay possible
Cross-regional support	Less	More

## Data Availability

Data are contained within the article.
